# Development and experimental validation of an osteoporosis diagnosis model based on disulfidoptosis-related genes and immune infiltration analysis

**DOI:** 10.3389/fimmu.2026.1834059

**Published:** 2026-05-28

**Authors:** Ping Yang, Minzhi Chen, Junxiang Zhang, Zhonghua Liu

**Affiliations:** 1 The National and Local Joint Engineering Laboratory of Animal Peptide Drug Development, College of Life Sciences, Hunan Normal University, Changsha, China; 2 Longevity Medicine Center, Hebei Boya Hospital, Baoding, China

**Keywords:** osteoporosis, disulfidoptosis, diagnostic model, immune infiltration, molecular subtypes

## Abstract

**Background:**

The role of disulfidoptosis, a novel form of programmed cell death, in osteoporosis remains unclear. This study aimed to identify disulfidoptosis-related molecular signatures for diagnosis, explore their link to immune infiltration, and validate their expression experimentally.

**Methods:**

We conducted an integrative bioinformatics analysis utilizing microarray datasets from the Gene Expression Omnibus (GEO). Analyses included the identification of disulfidoptosis-related differentially expressed genes (DRDEGs), weighted gene co-expression network analysis (WGCNA), and functional enrichment analyses. Three machine learning algorithms were employed to identify hub genes for diagnostic model construction. Immune infiltration patterns were assessed using CIBERSORT, and osteoporosis subtypes were identified via consensus clustering. Experimental validation was conducted in a RANKL-induced osteoclast model using qPCR and Western blot.

**Results:**

We identified 299 DRDEGs and constructed a diagnostic model based on three hub genes (SOAT2, FOLR3, TUBA8). The model showed moderate diagnostic accuracy in the internal validation set (AUC: 0.7-0.9) and high accuracy upon external validation (AUC > 0.9). Immune analysis revealed distinct infiltration patterns and significant correlations between key genes and immune cells. Consensus clustering defined two osteoporosis subtypes with distinct molecular and immune characteristics. Crucially, experimental validation confirmed significant dysregulation of these genes during osteoclast differentiation, with TUBA8 showing a striking discordance between mRNA upregulation and protein downregulation.

**Conclusion:**

This study establishes a diagnostic model for osteoporosis based on disulfidoptosis-related genes and provides multi-level experimental evidence for their dysregulation *in vitro*. Our findings may reveal immune heterogeneity and uncover potential post-transcriptional regulatory mechanisms, offering new insights for precision medicine in osteoporosis.

## 1 Introduction

Osteoporosis is a major global health concern and is characterized by reduced bone density and deterioration of bone microarchitecture, leading to greater fragility and higher fracture risk (1). It primarily affects the aging population, especially postmenopausal women and elderly men, and its prevalence continues to rise with global demographic shifts toward older age groups (2). Osteoporotic fractures, particularly of the hip, spine, and wrist, are associated with substantial morbidity, prolonged disability, and increased mortality. Additionally, the social and economic burdens imposed by such fractures are considerable, as reflected in higher healthcare costs, loss of independence, and diminished quality of life for the patients as well as their families (3).

Despite advances in understanding pathophysiology of osteoporosis and the development of pharmacologic agents that limit bone loss, challenges persist in the diagnosis and personalized management of osteoporosis. Currently available diagnostic methods, mainly dual-energy X-ray absorptiometry that measures bone mineral density, cannot fully capture bone quality or identify at-risk patients before they present with substantial bone deterioration (4). Moreover, although antiresorptive and anabolic therapies can reduce fracture risk, their efficacy is limited because of adverse effects, poor patient adherence, and the lack of biomarkers that can accurately predict treatment response or disease progression (5). Thus, there is an urgent need for diagnostic models that will not only enable early detection but also facilitate risk stratification and personalized intervention strategies.

Recently, programmed cell death (PCD) pathways have been implicated in regulating bone cell homeostasis (6). Disulfidoptosis, a newly identified PCD type triggered by disulfide stress under metabolic strain, has been associated with cancer and metabolic diseases (7, 8). Because bone cells are highly sensitive to metabolic and oxidative stress, disulfidoptosis may contribute to osteoporosis, although its role remains undefined. Prior studies have focused on traditional risk factors and bone metabolism–related genes (9), and there remains a paucity of studies systematically evaluating the diagnostic potential of disulfidoptosis-related genes (DRGs) in osteoporosis. Similarly, the link between expression of these genes and the immune landscape of osteoporotic bone tissue has not been clinically characterized. Defining these relationships could clarify the biological aspects of osteoporosis and reveal novel biomarkers for its early diagnosis and therapeutic targeting. Incorporating molecular and immunological parameters into diagnostic frameworks offers a promising strategy for advancing precision medicine in osteoporosis care.

This study aims to identify diagnostic features for osteoporosis based on disulfidoptosis-related genes, construct and validate a diagnostic model, and preliminarily investigate the potential biological significance of key genes by integrating immune-related analyses with *in vitro* experimental results.

## 2 Materials and methods

### 2.1 Data acquisition and preprocessing

This study incorporated three osteoporosis-related GEO datasets. GSE7158 (10) included 12 osteoporosis samples and 14 control samples, GSE56815 (11), included 40 osteoporosis samples and 40 control samples, and GSE230665 (12) included 12 osteoporosis samples and 3 control samples. Both GSE7158 and GSE56815 were derived from monocytes and were used for integrated discovery-stage analysis. including differential expression analysis, co-expression network construction, and feature gene screening; GSE230665 was obtained from femoral tissue and used as an independent external dataset for validating key genes and diagnostic models. Each dataset was assigned distinct functional roles within the study, with samples from different tissue sources not directly merged in a single modeling workflow.Basic information for each dataset is presented in **Table 1**.

**Table 1:** GEO microarray chip information.

GEO ID	GSE7158	GSE56815	GSE230665
Platform	GPL570	GPL96	GPL10332
Species	Homo sapiens	Homo sapiens	Homo sapiens
Tissue	Monocyte	Monocyte	Femur
Samples in Osteoporosis group	12	40	12
Samples in Control group	14	40	3
Reference	PMID: 19223260	PMID: 29330445	PMID: 37335694

GEO, Gene Expression Omnibus.

DRGs (Disulfidoptosis-related genes) were retrieved from the Genecards (13) database using the keyword “Disulfidoptosis", retaining only protein-coding genes as the candidate set. Additional DRGs were collected from PubMed (14–18) yielding 2, 870 unique genes after merging and deduplication (**Supplementary Table 1**). Considering the varying strength of evidence and research backgrounds of genes from different sources, this study utilized this gene set as a candidate pool for disulfidoptosis-related genes for subsequent screening and analysis. Genes directly derived from studies on disulfidoptosis-related were identified as core candidates, while genes obtained through database searches and literature expansion were classified as extended candidates. After merging and deduplicating genes from all sources, the total candidate gene set was generated and intersected with the gene expression matrix from the osteoporosis dataset resulted in 1, 832 DRGs for subsequent analysis (**Supplementary Table 2**).

Batch effects between GSE7158 and GSE56815 were corrected using the R package sva (v3.50.0), producing combined datasets of 52 osteoporosis and 54 control samples. Both the combined datasets and GSE230665 were normalized and annotated using the R package limma (v3.58.1). Principal component analysis (PCA) was used to verify effective batch effect removal.

The compilation of disulfidoptosis-related genes incorporated both database search results and literature-derived candidate genes, resulting in an initially broad gene set that maximally retained potential relevant signals during the exploratory phase, though it may have introduced some nonspecific background noise. Consequently, subsequent analyses did not draw conclusions directly based on all candidate genes. Instead, the candidate set was progressively narrowed through intersection analysis combined with differential expression analysis, co-expression network screening, and machine learning methods to enhance the robustness and interpretability of the results.

### 2.2 Identification of differentially expressed DRGs

Differentially expressed genes (DEGs) in the osteoporosis and control groups were analyzed using the R package limma, with |logFC|> 0 and P <0.05 serving as the screening criteria for differentially expressed genes. The differentially expressed genes were classified into upregulated and downregulated genes according to their logFC direction, and the results were visualized using a volcano plot. Subsequently, the intersection between the differentially expressed genes (DEGs) and the disulfidoptosis-related genes (DRGs) set was identified to obtain the Disulfidoptosis-Related Differentially Expressed Genes (DRDEGs), which were then represented by a Venn diagram and a heat map to illustrate their expression patterns.

The screened DRDEGs were further utilized for subsequent co-expression network analysis, feature gene identification, and diagnostic model construction. The relevant results were comprehensively evaluated by integrating a multi-step analytical workflow.

### 2.3 Weighted gene co-expression network analysis

Using the R package WGCNA (v1.72.5), weighted gene co-expression network analysis (WGCNA) was performed on the top 20% most variable genes from the combined datasets. The soft threshold power was set to 4, with a scale-free fit index of 0.90. Modules were identified with a minimum of 25 genes and a merge cut height of 0.6. Modules with |r value| > 0.3 were selected for further analysis. Genes overlapping between the most relevant module and DRDEGs were used for further analysis.

Prior to constructing the WGCNA network, genes in the integrated dataset were pre-screened based on gene expression variance, with the top 20% of genes by variance selected for subsequent analysis. This step aims to retain genes with higher expression variability and relatively richer information content, while minimizing interference from low-variability genes in the construction of co-expression networks, thereby enhancing the stability and interpretability of module identification. Subsequently, a weighted gene co-expression network was constructed on this basis to identify key modules associated with the osteoporosis phenotype.

### 2.4 Functional enrichment analysis

The R software package clusterProfiler (v4.10.0) was used for Gene Ontology (GO) analysis and Kyoto Encyclopedia of Genes and Genomes (KEGG) enrichment analyses of genes from the most correlated module, with significance defined as P< 0.05.

### 2.5 Gene set enrichment analysis

Gene set enrichment analysis (GSEA) evaluates whether genes from a specified gene set are overrepresented at the top or bottom of a ranked gene list associated with a phenotype. GSEA was performed on all genes in the combined datasets using the R package clusterProfiler and the c2.all.v2024.1.Hs.symbols.gmt gene set from the Molecular Signatures Database. Parameters included 1, 000 permutations, a seed of 2022, and gene set size of 10–500. Significance was defined as adjusted P < 0.05 and false discovery rate (FDR) < 0.25.

### 2.6 Machine learning–based diagnostic model construction

Three machine learning algorithms, namely support vector machine (SVM-RFE), least absolute shrinkage and selection operator (LASSO), and extreme gradient boosting (XGB), were applied to module genes to identify key genes for osteoporosis diagnosis. SVM-RFE was implemented using the R package e1071 (v1.7-14), LASSO regression using the R package glmnet (v4.1-8) with family = “binomial, “ and XGB using the R package xgboost with five-fold cross-validation. Final key genes were defined as those selected by all three methods.

### 2.7 Internal and external validation of the diagnostic model

The diagnostic model was tested on the combined datasets (internal validation) and GSE230665 (external validation). Receiver operating characteristic (ROC) curves were plotted using the R package pROC (v1.18.5), and area under the curve (AUC) values were calculated. AUC for ROC curves range from 0.5 to 1.0, with 0.5–0.7 indicating low diagnostic accuracy, 0.7–0.9 moderate accuracy, and >0.9 high accuracy. Nomograms and calibration curves were generated, and decision curve analysis (DCA) performed, using the R packages rms (v6.7.1) and ggDCA (v1.1) to assess model performance and clinical utility.

### 2.8 Consensus clustering for osteoporosis subtyping

Consensus clustering was performed via the R package ConsensusClusterPlus (v1.62.0) using key gene expression profiles to define osteoporosis subtypes. Parameters included 1, 000 iterations, 95% resampling, and Pearson distance. The optimal cluster number was determined as 2–10.

### 2.9 Gene set variation analysis

Gene set variation analysis (GSVA) is a nonparametric, unsupervised analytical method that transforms a gene expression matrix into a gene set expression matrix for evaluation of enrichment across samples. GSVA was performed using the R package GSVA with the c2.cp.v2023.2.Hs.symbols.gmt gene set to examine pathway activity differences between osteoporosis subtypes. Significance was defined as P < 0.05.

### 2.10 Immune infiltration analysis

CIBERSORT is a deconvolution algorithm based on linear support vector regression that estimates immune cell composition from bulk transcriptomic data. The CIBERSORT algorithm was employed in conjunction with a reference immune cell expression feature matrix to perform deconvolution analysis on bulk transcriptomic data, thereby estimating the relative compositional characteristics of 22 immune cell types in the samples. Subsequently, correlation analyses were conducted on the predicted immune cell-related results, and their associations with immune-related features were evaluated by examining key gene expression patterns.Correlations between key genes and immune cells were tested using Spearman’s method and displayed as heatmaps and bubble plots.

### 2.11 Cell culture and osteoclast differentiation model

The murine RAW264.7 cells (Procell, Wuhan, China) were cultured in Dulbecco’s Modified Eagle Medium (DMEM; Pricella, PM150210, Wuhan, China) supplemented with 10% fetal bovine serum (FBS; Pricella, 164210, Wuhan, China) and 1% penicillin/streptomycin (Solarbio, P1400, Beijing, China). Cells were maintained in a humidified incubator (LiShen, HF151, Shanghai, China) at 37 °C with 5% CO_2_.

To induce osteoclast differentiation, cells were seeded in 6-well plates (Corning, 3516, USA) at an appropriate density and treated with 50 ng/mL receptor activator of nuclear factor kappa-B ligand (RANKL; MCE, HY-P73388, USA) for 6 days to establish the model group. The culture medium was refreshed every 3 days. Cells maintained in standard medium without RANKL served as controls. Morphological changes were monitored using an inverted microscope (Sunny, XD-RFL, Ningbo, China), and cells were harvested for subsequent analyses.

### 2.12 Quantitative real-time PCR validation

Total RNA was extracted from RAW264.7 cells using a commercial RNA extraction kit (Solarbio, R1200, Beijing, China) according to the manufacturer’s instructions. RNA concentration and purity were assessed using a spectrophotometer (Lifereal, F-1100, Hangzhou, China). cDNA was synthesized from 1000 ng of total RNA using a reverse transcription kit (Hifair^®^ III SuperMix Plus, Yeasen, 11141ES60, Shanghai, China). Quantitative real-time PCR (qPCR) was performed on a MA-6000 system (Molarray, Suzhou, China) using SYBR Green Master Mix (Yeasen, 11201ES08, Shanghai, China). The PCR conditions were as follows: 95 °C for 5 min, followed by 40 cycles of 95 °C for 10 s, 60 °C for 20 s, and 72 °C for 20 s. The 2–ΔΔCt method was used to calculate relative gene expression levels, with GAPDH as the internal control. Primer sequences are listed in **Table 2**.

**Table 2:** Primer sequences.

Gene	Forward primer	Reverse primer
GAPDH(m)	GTGAAGGTCGGTGTGAACGGATT	CGTGAGTGGAGTCATACTGGAACAT
M-SOAT2	TGCTGCTCTCCATCTTGCAT	ACATCCTGTCTCCAAACCGC
M-OR10H1	CACACTTCACTCCATCGCCT	AGAGGTGGAACATGACAAGCA
M-KCNF1	AAGGGCAGTTAAAGGACGGG	ACAAGGCTCTACGGCTCCTA
M-TUBA8	ACCTGGATATTGAACGCCCC	GCTCGTGGTAGGCTTTCTCA

Despite multiple primer design attempts, specific amplification of murine FOLR3 mRNA could not be achieved; This result may be attributed to species-specific differences between FOLR3 in humans and mice, as well as the absence of a clearly defined genetic background in the murine model system; consequently, this study failed to obtain valid validation at the mRNA level.

### 2.13 Western blot analysis

RAW264.7 cells were lysed in RIPA buffer (Beyotime, P0013B, Shanghai, China) supplemented with protease and phosphatase inhibitors (Beyotime, P1045, Shanghai, China). Protein concentrations were determined using a bicinchoninic acid assay (BCA) (Fubio, BL521, suzhou, China). Equal amounts of protein (20 μg per lane) were separated by 10% SDS-PAGE and transferred to PVDF membranes (Millipore, Bedford, MA, USA). Membranes were blocked with 5% non-fat milk for 2h at room temperature (20–25 °C) and incubated overnight at 4 °C with primary antibodies against FOLR3 (BD-PN0716, 1:2000, Biodragon, Beijing, China), KCNF1 (BD-PT2453, 1:2000, Biodragon, Beijing, China), OR10H1 (BD-PT3254, 1:2000, Biodragon, Beijing, China), SOAT2 (BD-PN7276, 1:2000, Biodragon, Beijing, China), TUBA8 (BD-PE4160, 1:2000, Biodragon, Beijing, China), and GAPDH (10494-1-AP, 1:5000, Proteintech, Wuhan, China). After washing, membranes were incubated with HRP-conjugated secondary antibodies (bs-0295G-HRP, 1:10000, Bioss, Beijing, China) for 1 h at room temperature (20–25 °C). Protein bands were visualized using an enhanced chemiluminescence (ECL) reagent (Fubio, F044, Suzhou, China) and imaged with a chemiluminescence imaging system (Tanon, 5200, Beijing, China). Band intensities were quantified using ImageJ software. Data are presented as the mean ± standard deviation (SD) from three independent biological replicates, each with three technical replicates.

### 2.14 Statistical analysis

All analyses were conducted using the R software (v4.3.0). Group comparisons were conducted using Student’s t-test or the Mann–Whitney U test for two groups and the Kruskal–Wallis test for multiple groups. Experimental data are presented as mean ± SD from at least three independent experiments. Spearman correlation was used for association testing. A two-sided P value of <0.05 was considered statistically significant.

## 3 Results

### 3.1 Study flow chart

**Figure 1:**
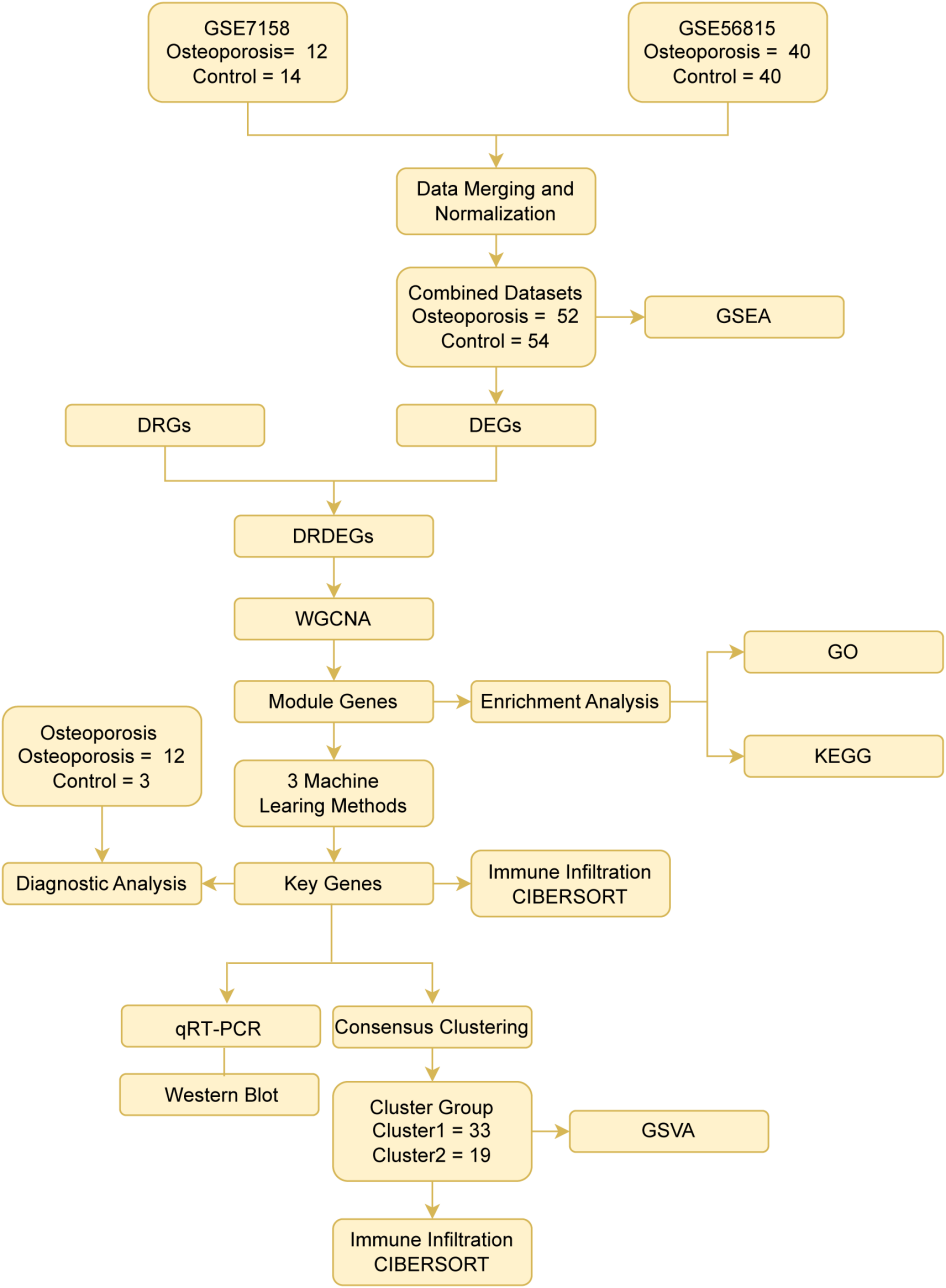
Flow chart for comprehensive DRDEG analysis. GSEA, gene set enrichment analysis; DEGs, differentially expressed genes; DRGs, disulfidoptosis-related genes; GO, Gene Ontology; KEGG, Kyoto Encyclopedia of Genes and Genomes; DRDEGs, disulfidoptosis-related differentially expressed genes; WGCNA, weighted correlation network analysis; GSVA, gene set variation analysis.

The study design flow chart is presented in **Figure 1**.

### 3.2 Data preprocessing and batch effect removal

**Figure 2:**
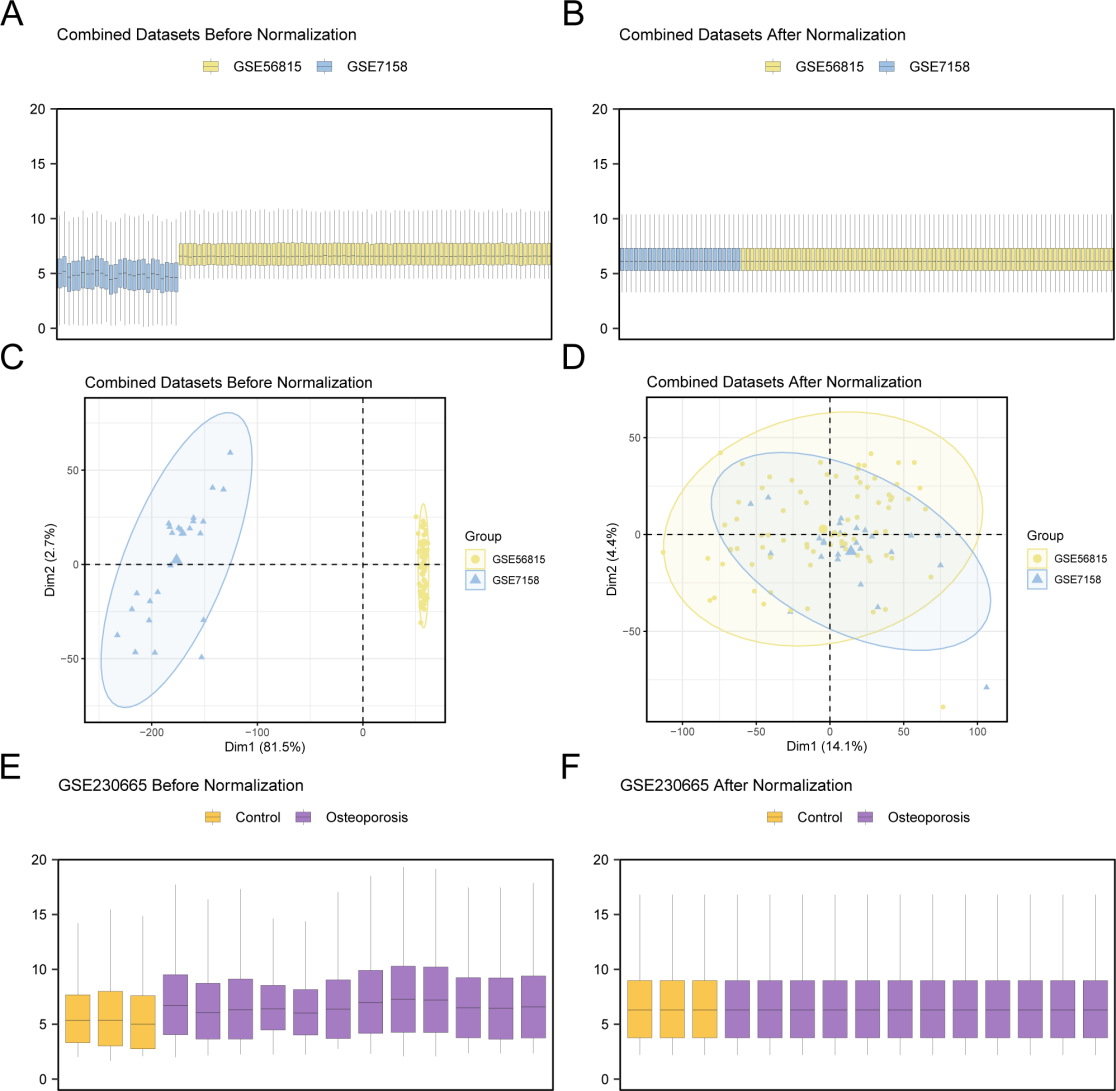
Batch effect removal of combined datasets and GSE230665. **(A)** Boxplot of combined datasets before batch correction. **(B)** Boxplot of combined datasets after batch correction. **(C)** Principal component analysis (PCA) plot of combined datasets before correction. **(D)** PCA plot of combined datasets after correction. Datasets GSE56815 and GSE7158 are shown in yellow and blue, respectively. **(E)** Boxplot of GSE230665 before normalization. **(F)** Boxplot of GSE230665 after normalization. Osteoporosis and control samples are shown in purple and orange, respectively.

Two osteoporosis datasets (GSE7158 and GSE56815) were integrated after batch effect correction using the sva package. Boxplots and PCA confirmed effective correction, as shown by improved sample clustering (**Figures 2A–D**). The GSE230665 dataset was normalized and annotated using the limma package, with boxplots confirming standardized distributions (**Figures 2E, F**).

### 3.3 Identification of disulfidoptosis-related DEGs

**Figure 3:**
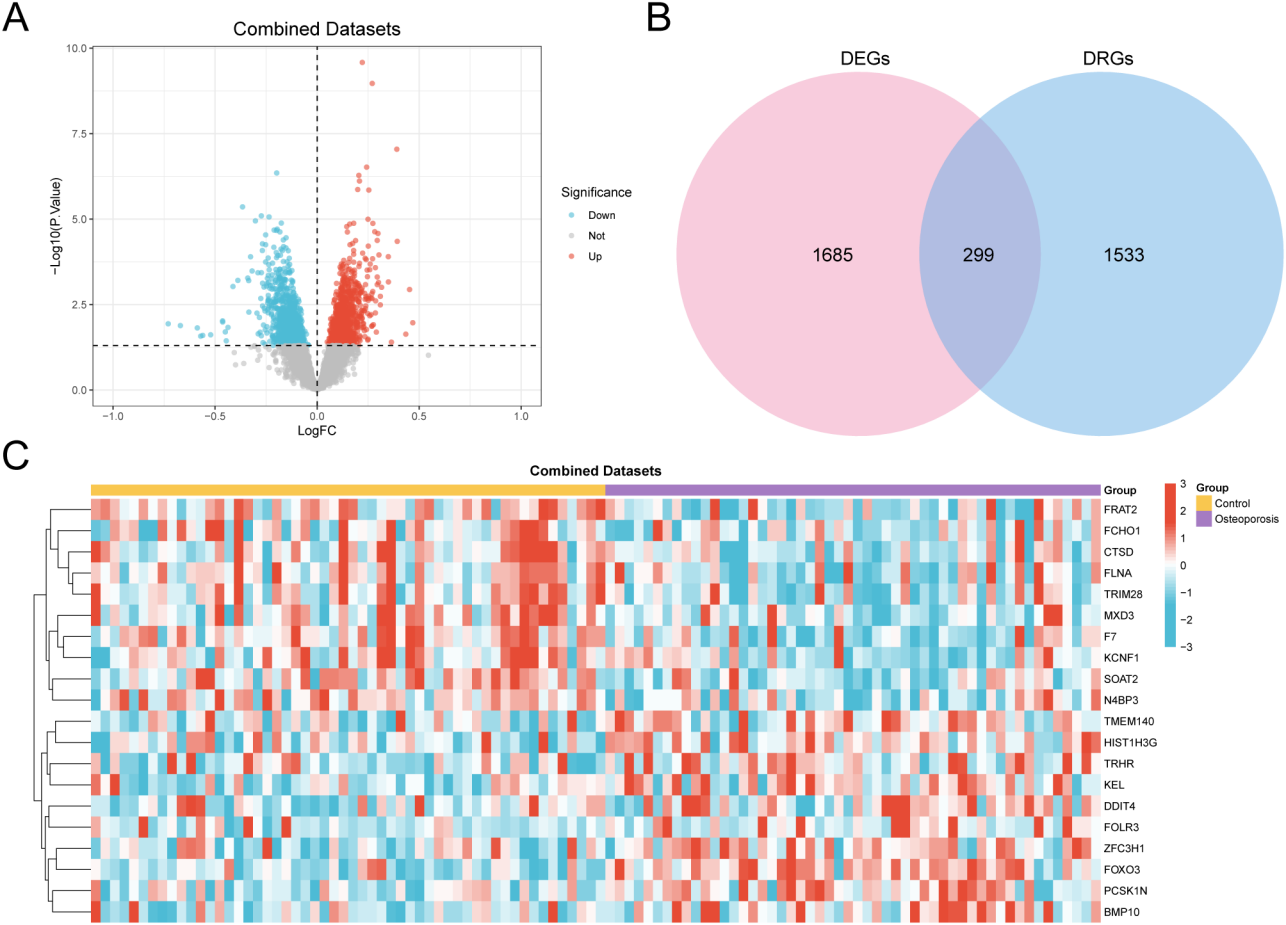
Differential gene expression analysis. **(A)** Volcano plot of DEGs in the combined GEO datasets. **(B)** Venn diagram of DEGs and DRGs in the combined GEO datasets. **(C)** Heat map of DRDEGs in the combined GEO datasets. DEGs, differentially expressed genes; DRGs, disulfidoptosis-related genes; DRDEGs, disulfidoptosis-related DEGs. Osteoporosis and control groups are shown in purple and orange, respectively. In the heat map, red and blue represent high and low expression levels, respectively.

Differential expression analysis between the osteoporosis and control groups identified 1, 984 DEGs (|logFC| > 0, P < 0.05), including 991 and 993 upregulated and downregulated genes, respectively (**Figure 3A**). Intersection with 1, 832 DRGs yielded 299 DRDEGs (**Figure 3B**; **Supplementary Table 3**). A heatmap of the top 20 DRDEGs revealed distinct expression differences between groups (**Figure 3C**).

### 3.4 WGCNA

**Figure 4:**
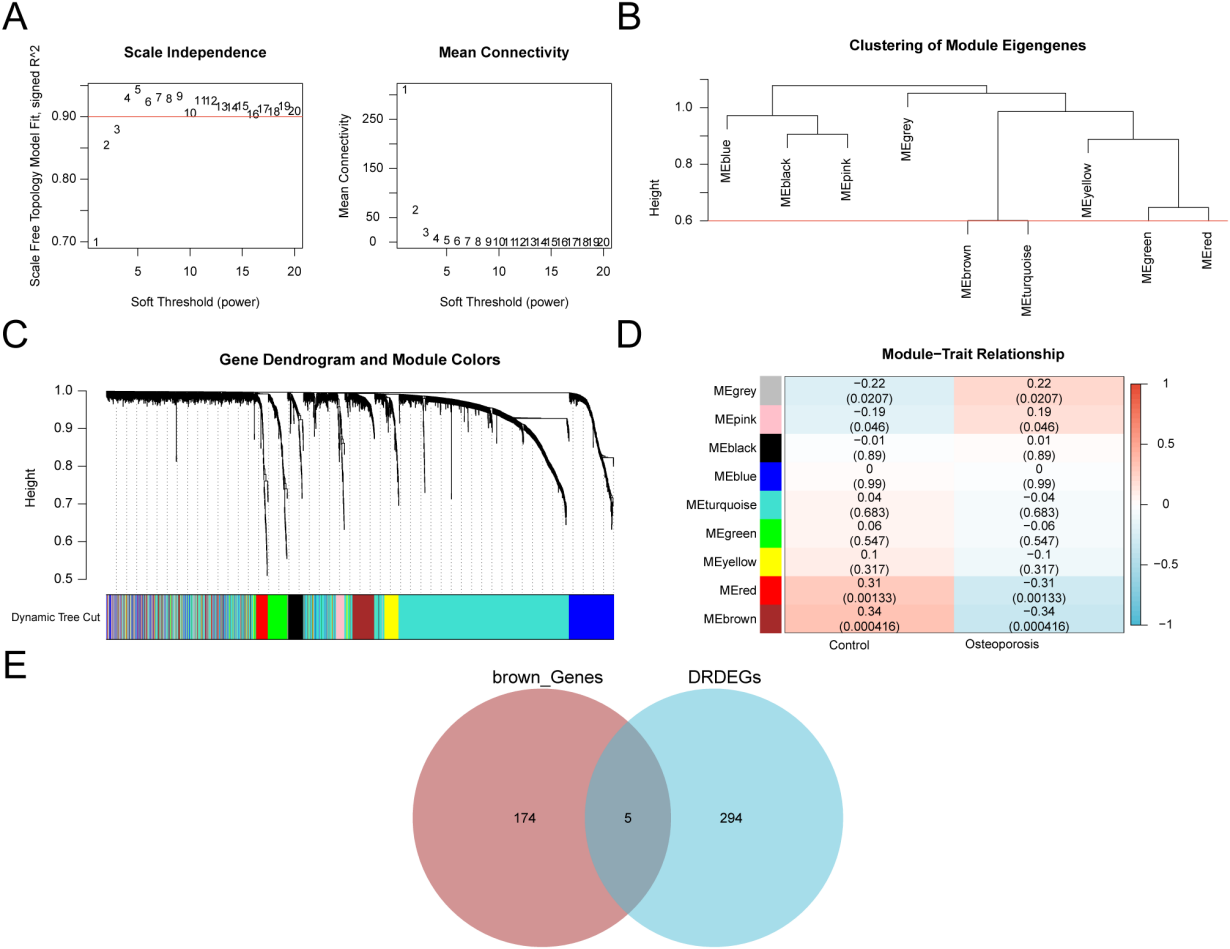
WGCNA for the combined datasets. **(A)** Scale-free network showing optimal soft threshold (left) and network connectivity at different thresholds (right). **(B)** Module clustering of the top 20% variable genes. **(C)** Hierarchical clustering dendrogram (top) and gene module assignment (bottom). **(D)** Correlation between gene modules and osteoporosis/control groups. **(E)** Venn diagram of DRDEGs and MEbrown genes. Correlation coefficients (r values) < 0.3 denote weak/no correlation, whereas 0.3–0.5 denotes weak correlation. Red and blue represent positive and negative correlations, respectively.

WGCNA identified nine coexpression modules from the top 20% most variable genes: MEgrey, MEpink, MEblack, MEblue, MEturquoise, MEgreen, MEyellow, MEred, and MEbrown. A soft threshold of 4 was set to achieve a scale-free topology (**Figure 4A**). The MEbrown module correlated most strongly with osteoporosis (|r| > 0.3) and contained 179 genes (**Figures 4B–D**; **Supplementary Table 4**). Overlap with DRDEGs yielded five module genes: folate receptor 3 (FOLR3), OR10H1, KCNF1, sterol O-acyltransferase 2 (SOAT2), and tubulin alpha 8 (TUBA8) (**Figure 4E**).

It should be noted that the module selection threshold employed in this study within WGCNA is exploratory in nature. The identified modules reflect co-expression patterns exhibiting certain correlations with the osteoporosis phenotype, rather than strong statistical correlations. Therefore, the biological significance of these modules should be comprehensively interpreted through subsequent intersection screening, machine learning analysis, external validation, and experimental results.

### 3.5 Enrichment analysis of module genes from functional and GSEA perspectives

**Figure 5:**
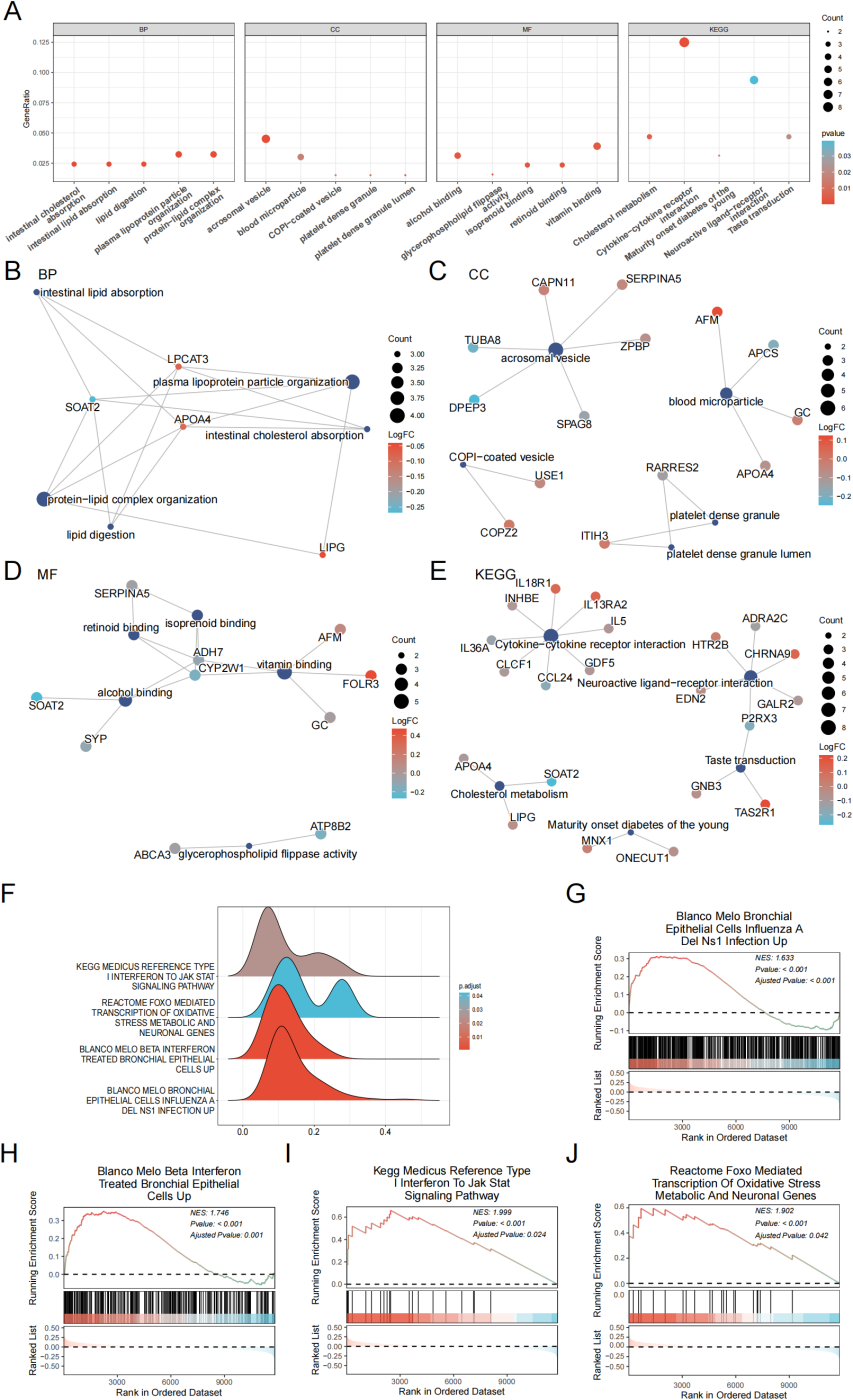
Functional enrichment analysis of MEbrown module genes and gene set enrichment analysis (GSEA) based on combined datasets. **(A)** Bubble map of Gene Ontology (GO) enrichment for 179 genes in the MEbrown module showing biological process (BP), cellular component (CC), molecular function (MF) and biological pathway (KEGG) categories. Network diagrams of enrichment results: BP **(B)**, CC **(C)**, MF **(D)**, and KEGG **(E)**. Dark blue nodes represent entries, and lines represent the relationships between entries and molecules. In molecular nodes, red and blue represent upregulation and downregulation, respectively. Bubble size and color in the bubble plot represent the number of genes and P value size, respectively (red, smaller P value; blue, larger P value). Screening threshold for GO and KEGG enrichment analyses was P < 0.05. **(F)** Overview of four biological functions in osteoporosis datasets. **(G–J)** GSEA showed significant enrichment in the following: Blanco–Melo bronchial epithelial cells influenza A Del Ns1 infection **(G)**; Blanco–Melo β-interferon–treated bronchial epithelial cells **(H)**; Medicus reference type I interferon to Jak–Stat signaling **(I)**; Foxo-mediated transcription of oxidative stress, metabolic, and neuronal genes **(J)**. Screening criteria for GSEA: adjusted P < 0.05 and FDR (q value) < 0.25, with Benjamini–Hochberg correction.

GO and KEGG enrichment of the 179 MEbrown genes linked osteoporosis to multiple processes and pathways (**Supplementary Table 5**). Enriched biological processes (BPs) included intestinal cholesterol absorption in osteoporosis, plasma lipoprotein particle organization, lipid digestion, intestinal lipid absorption, and protein–lipid complex organization. Enriched cellular components (CCs) included acrosomal vesicle, platelet dense granule lumen, platelet dense granule, COPI-coated vesicle, and blood microparticle. Enriched molecular functions (MFs) included retinoid binding, isoprenoid binding, alcohol binding, glycerophospholipid flippase activity, and vitamin binding. KEGG pathways included cytokine–cytokine receptor interaction, cholesterol metabolism, maturity-onset diabetes of the young, taste transduction, and neuroactive ligand–receptor interaction. Results were visualized via bubble plots (**Figure 5A**). GO and KEGG enrichment networks were also constructed (**Figures 5B–E**).

GSEA of all genes in the combined datasets identified significant enrichment in interferon response, immune-related, and oxidative stress pathways (**Figures 5F–J**; **Supplementary Table 6**), including Blanco–Melo bronchial epithelial cell infection and Foxo-mediated transcription pathways.

### 3.6 Machine learning–based identification of key genes

**Figure 6:**
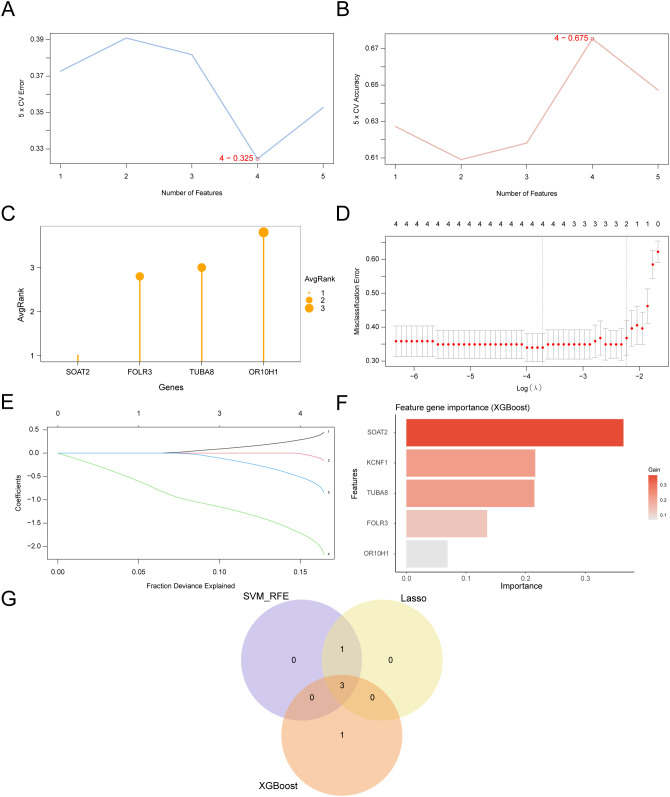
Screening of key osteoporosis genes via machine learning. A, **(B)** Number of genes with the lowest error rate **(A)** and highest accuracy **(B)** from SVM-RFE modeling. **(C)** Lollipop plot of average importance ranking for the four lowest-error genes based on SVM-RFE modeling. **(D, E)** Diagnostic model plot **(D)** and variable trajectory plot **(E)** of LASSO regression. **(F)** Top four genes ranked by XGB-modeling importance. **(G)** Venn diagram of genes screened by all three machine learning algorithms. SVM-RFE, support vector machine recursive feature elimination; LASSO, least absolute shrinkage and selection operator; XGB, extreme gradient boosting.

The machine learning algorithms SVM-RFE, LASSO, and XGB were applied to the five module genes. SVM-RFE and LASSO both identified the genes SOAT2, FOLR3, TUBA8, OR10H1, whereas XGB prioritized SOAT2, KCNF1, TUBA8, and FOLR3 (**Figures 6A–F**). Thus, their intersection yielded three key genes: SOAT2, FOLR3, and TUBA8 (**Figure 6G**).

### 3.7 Internal and external validation of the diagnostic model

**Figure 7:**
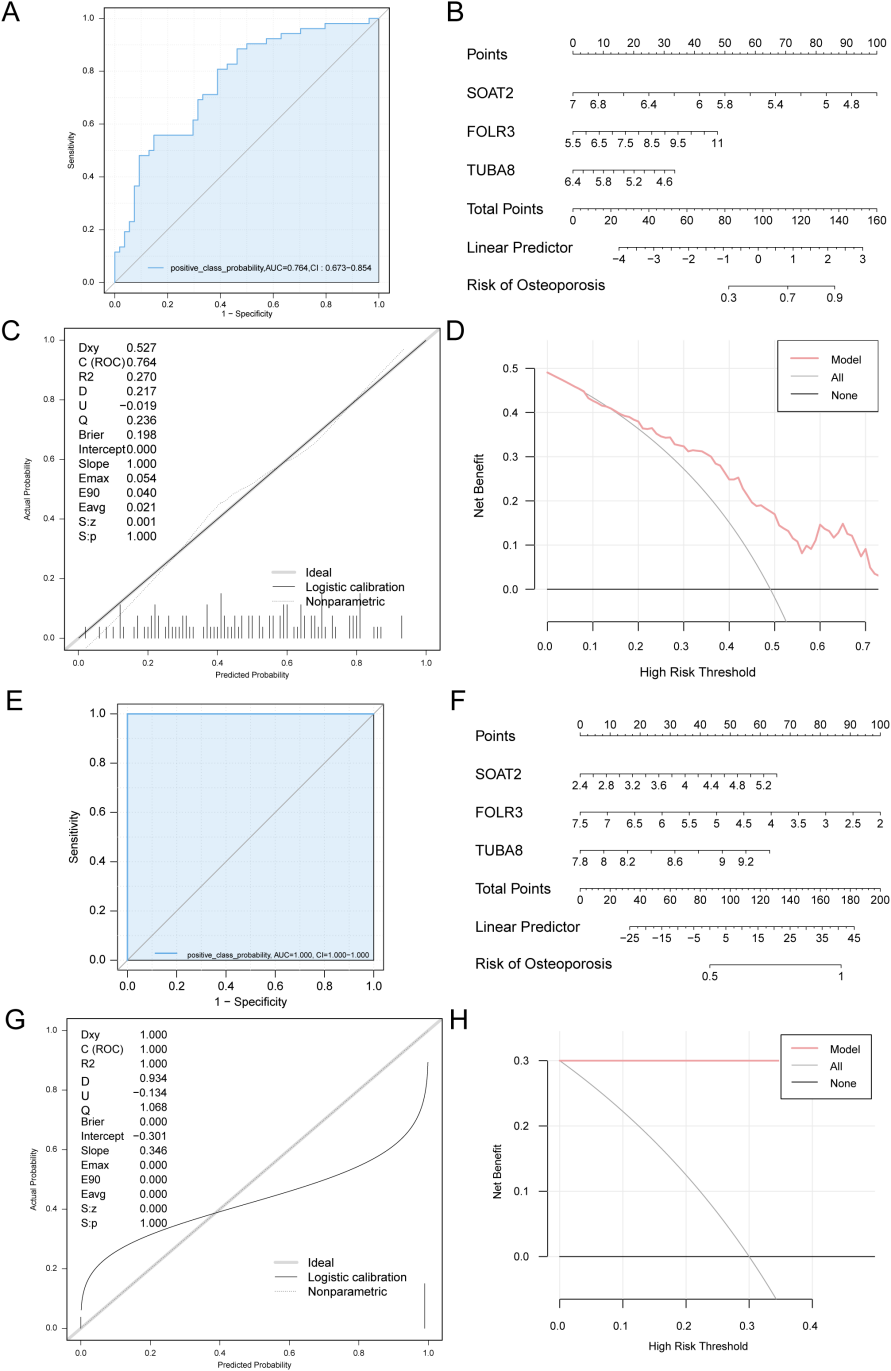
Internal and External Validation of the Diagnostic Model. **(A-D)** Internal validation of the diagnostic model. **(A)** ROC curves of the logistic regression model in osteoporosis and control samples in combined GEO datasets. **(B)** Nomograms of key genes in the combined datasets in osteoporosis diagnostic models. **(C, D)** Calibration curve plot **(C)** and DCA plot **(D)** of key genes in the combined datasets in osteoporosis diagnostic models. An AUC of 0.7–0.9 indicates moderate accuracy. E-H. External validation of the diagnostic model. **(E)** ROC curve of logistic regression in osteoporosis and control samples in GSE230665. **(F)** Nomogram of key genes in GSE230665. **(G, H)** Calibration curve **(G)** and DCA plot **(H)** of key genes in GSE230665. DCA, decision curve analysis; ROC, receiver operating characteristic; AUC, area under the curve. AUC > 0.9 indicates high accuracy.

A logistic regression model based on the three key genes achieved an AUC of 0.7–0.9 in the combined dataset, indicating moderate accuracy (**Figure 7A**). A nomogram highlighted SOAT2 as the most influential gene (**Figure 7B**). Calibration and decision curve analyses confirmed the model’s reliability and clinical utility (**Figures 7C, D**). External validation using the GSE230665 dataset showed high diagnostic accuracy (AUC > 0.9; **Figure 7E**). Nomogram and calibration curves further supported model robustness (**Figures 7F–H**).

In the GSE230665 dataset, models constructed based on key genes demonstrated superior discriminative power. Given that this dataset originates from femoral tissue while the models were built using monocyte-derived data, these findings are particularly suitable as supportive validation signals across diverse biological contexts, indicating that relevant genetic features retain discernment capability across tissue datasets. However, their performance should be interpreted with caution considering variations in tissue sources.

### 3.8 Immune infiltration analysis in osteoporosis versus controls via CIBERSORT

**Figure 8:**
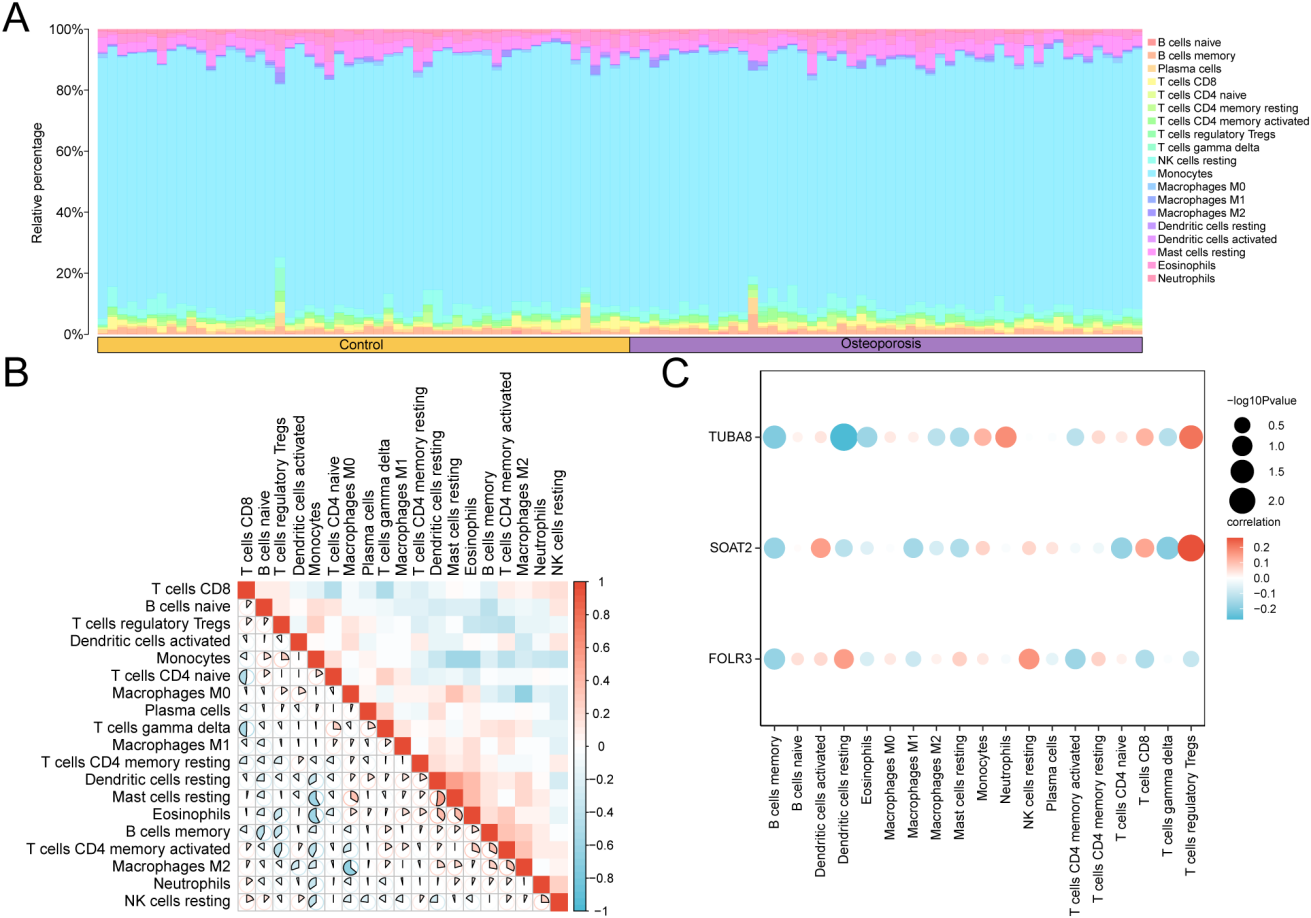
Immune infiltration analysis of combined datasets via CIBERSORT. **(A)** Bar chart of immune cell proportions in combined GEO datasets. **(B)** Heat map of immune cell relevance in combined GEO datasets. **(C)** Bubble plot of key gene correlations with immune cell infiltration in combined GEO datasets. Correlation coefficients (r values) < 0.3 indicate weak/no correlation, 0.3–0.5 weak correlation, and 0.5–0.8 moderate correlation. Orange and purple represent the control and osteoporosis groups, respectively. Red and blue show positive and negative correlations, respectively. Color represents correlation strength.

Based on CIBERSORT deconvolution analysis, the integrated dataset revealed distinct immune cell-related characteristic differences among various sample groups (**Figure 8A**). Correlation analysis showed significant associations between key genes and immune cells, e.g. SOAT2 correlated positively with Tregs, whereas TUBA8 correlated negatively with resting dendritic cells (**Figures 8B, C**). These findings reflect potential immune composition alterations in the osteoporosis-associated transcriptomic context and their possible associations with key genes.

### 3.9 Identification of osteoporosis subtypes and GSVA analysis

**Figure 9:**
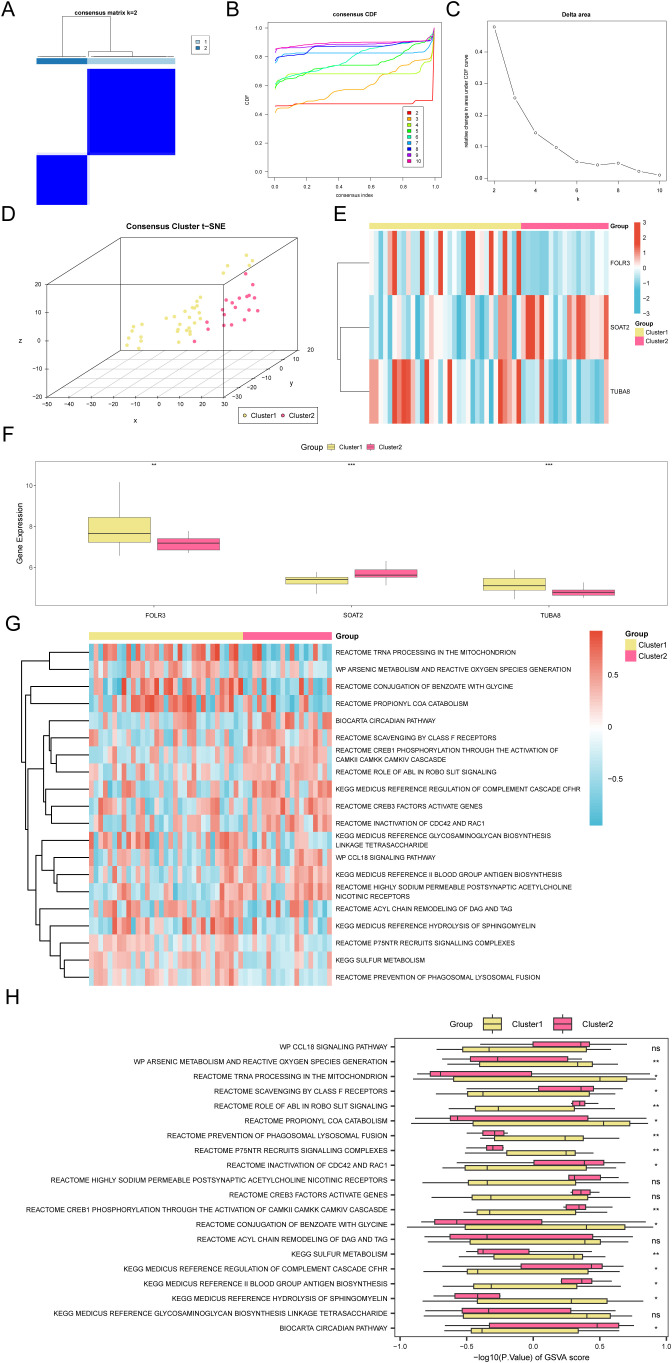
Consensus clustering analysis and gene set variance analysis (GSVA) of osteoporosis subtypes. **(A)** Consensus clustering plots of osteoporosis samples in combined GEO datasets. **(B, C)** CDF plot **(B)** and Delta plot **(C)** of consistency clustering. **(D)** Three-dimensional t-SNE cluster map of two osteoporosis subtypes. **(E)** Heat map of key gene expression in osteoporosis subtypes. **(F)** Group comparison of key genes between osteoporosis subtypes. CDF, cumulative distribution function; t-SNE, t-distributed stochastic neighbor embedding. **P < 0.01 and ***P < 0.001. Yellow and pink represent the subtypes Cluster 1 and Cluster 2, respectively. In the heatmap, red and blue show upregulation and downregulation, respectively.**(G, H)** Heatmap **(G)** and group comparison map **(H)** for GSVA of osteoporosis subtypes in the combined GEO datasets. GSVA, gene set variation analysis; ns, P ≥ 0.05 (not statistically significant); *P < 0.05 and **P < 0.01. Yellow and pink represent subtypes Cluster 1 and Cluster 2, respectively. Blue and red represent low and high enrichment, respectively, in the heatmap. GSVA screening threshold was P < 0.05.

Consensus clustering of key gene expression identified two osteoporosis subtypes termed Cluster 1 (n = 33) and Cluster 2 (n = 19) (**Figures 9A–D**). Heatmap and boxplot analyses confirmed significant SOAT2, FOLR3, and TUBA8 expression differences between these subtypes (**Figures 9E, F**).

GSVA revealed significant pathway activity differences between osteoporosis subtypes, including enriched pathways for tRNA processing in mitochondria and scavenging by class F receptors, along with depleted pathways for arsenic metabolism and sulfur metabolism (**Figures 9G, H**; **Supplementary Table 7**).

### 3.10 Distinct immune patterns between osteoporosis molecular subtypes

**Figure 10:**
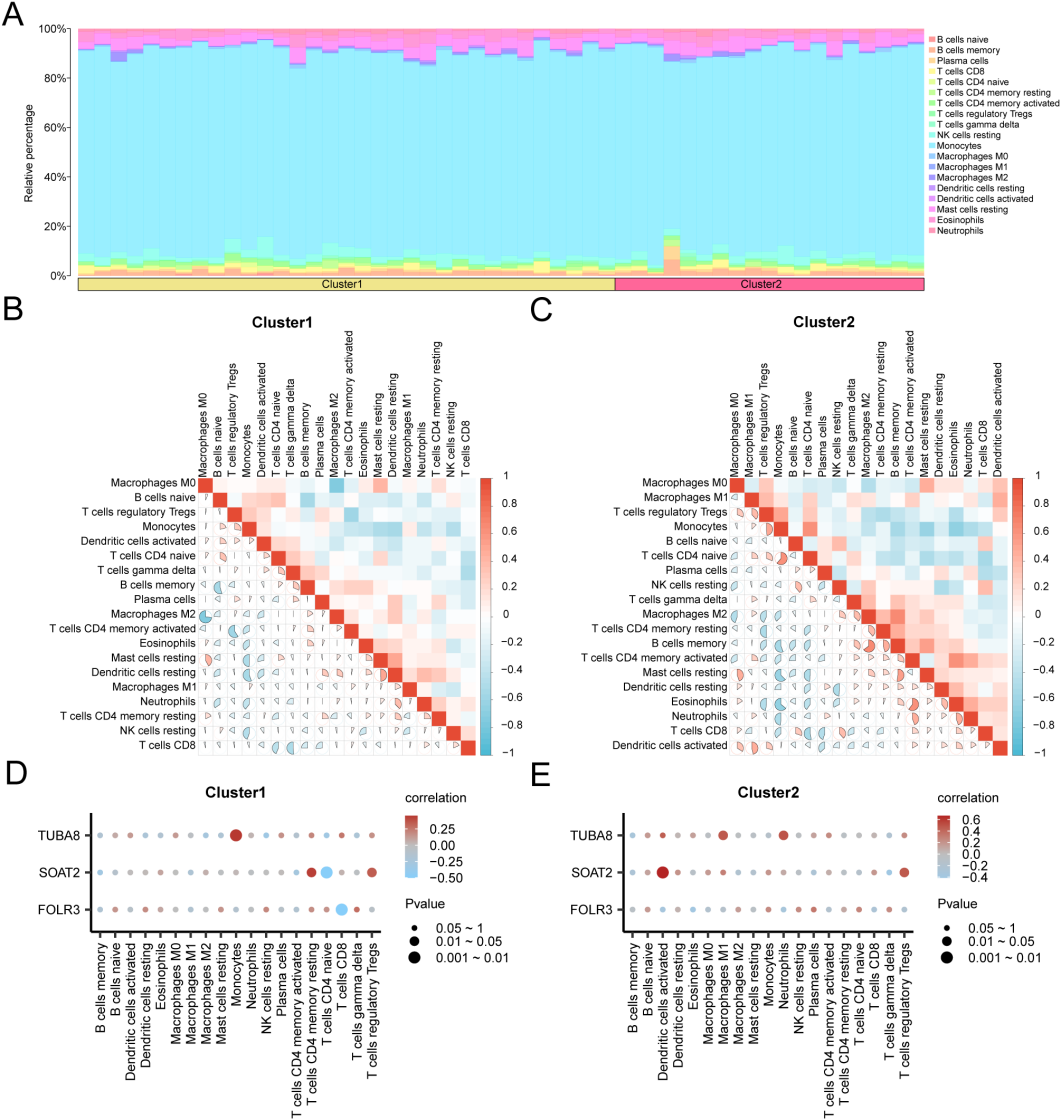
Immune infiltration analysis via CIBERSORT in osteoporosis subtypes. **(A)** Bar chart of immune cell proportions in osteoporosis subtypes **(A)**. **(B, C)** Correlation heatmaps of immune cells in the subtypes Cluster 1 **(B)** and Cluster 2 **(C)** of osteoporosis samples. **(D, E)** Bubble plot of key gene correlations with immune cell infiltration in the subtypes Cluster 1 **(D)** and Cluster 2 **(E)** of osteoporosis samples. Correlation coefficients (r values) < 0.3 indicate weak/no correlation, 0.3–0.5 weak correlation, and 0.5–0.8 moderate correlation. Yellow and pink represent the subtypes Cluster 1 and Cluster 2, respectively. Red and blue indicate positive and negative correlations, respectively. Color depth represents correlation strength.

Immune cell composition differed between osteoporosis subtypes (**Figure 10A**). Correlation analyses within subtypes revealed distinct immune cell–gene interactions, e.g., TUBA8 correlated with monocytes in Cluster 1, whereas SOAT2 correlated with activated dendritic cells in Cluster 2 (**Figures 10B–E**).

Among the different molecular subtypes of osteoporosis, CIBERSORT prediction results reveal distinct differences in immune cell-related characteristics, with varying correlation patterns observed between key genes and certain immune cell features within each subtype. These findings suggest that different molecular subtypes may exhibit heterogeneous immune-related expression profiles.

### 3.11 Experimental validation of key gene expression via qPCR and western blot

**Figure 11:**
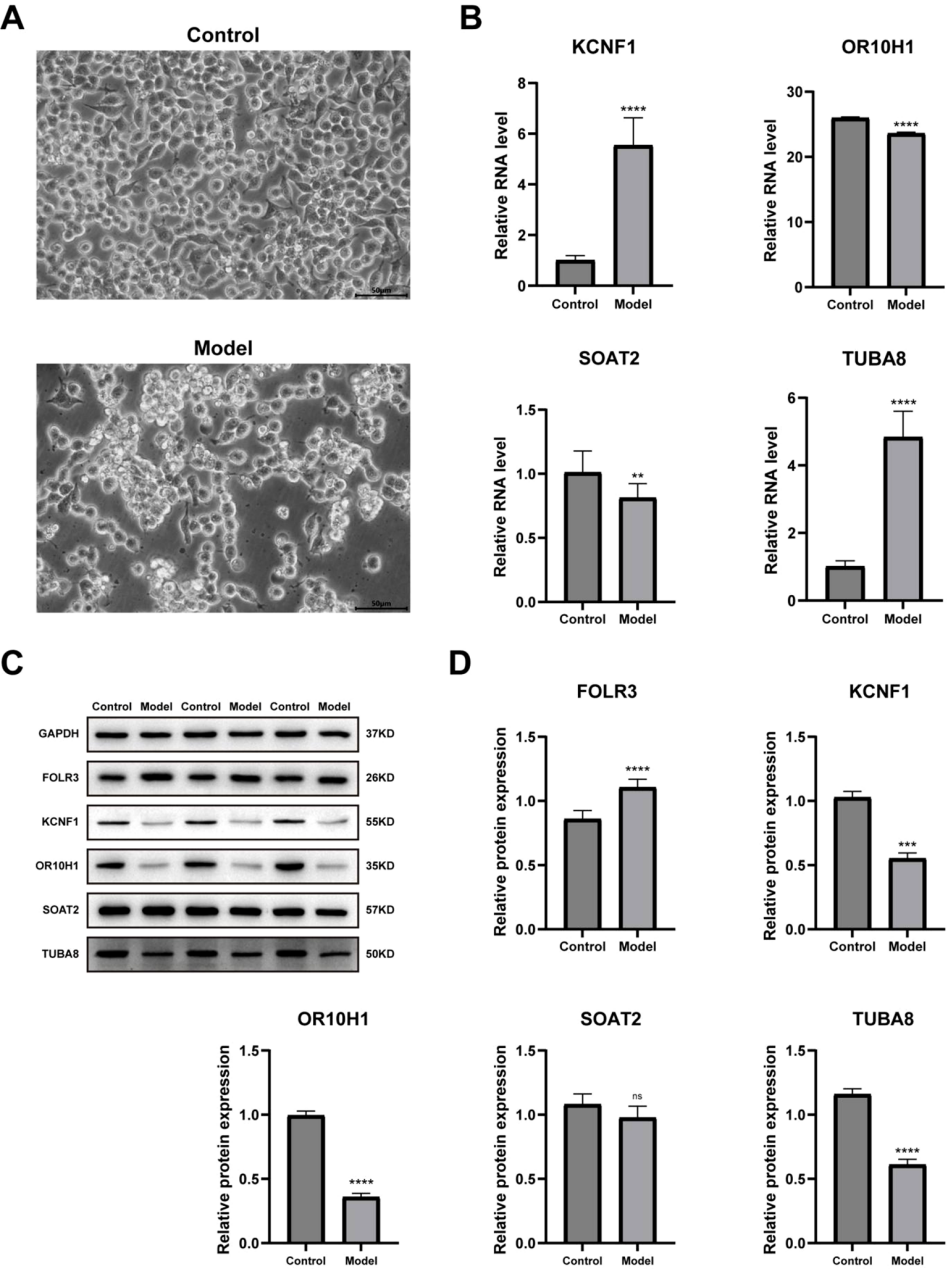
Experimental validation of key gene expression in a RANKL-induced osteoclast differentiation model. **(A)** Representative micrographs showing morphological changes of RAW264.7 cells after 6 days of RANKL (50 ng/mL) induction. Scale bar = 50 μm. **(B)** mRNA expression levels of SOAT2, TUBA8, OR10H1, and KCNF1 detected by qPCR. GAPDH was used as an internal control. The mRNA level of FOLR3 was not determined (N.D.). **(C)** Quantitative densitometric analysis of western blot results. **(D)** Representative western blot images showing protein expression levels of FOLR3, KCNF1, OR10H1, SOAT2, and TUBA8. GAPDH was used as a loading control (n=3).*P < 0.05, **P < 0.01, ***P < 0.001, ****P < 0.0001, ns, not significant.

To biologically validate our bioinformatics predictions, we examined the expression of the five candidate genes (SOAT2, FOLR3, TUBA8, OR10H1, and KCNF1) in a RANKL-induced osteoclast differentiation model using RAW264.7 cells. Morphological observations confirmed successful model establishment. Compared with the control group, RAW264.7 cells treated with RANKL for 6 days progressively fused to form large, multinucleated osteoclasts, accompanied by a significant increase in cell volume and the appearance of pseudopod-like structures at the cell periphery (**Figure 11A**).

At the mRNA level, quantitative real-time PCR (qPCR) was performed for four of the five genes. Consistent with our computational findings, the expression levels of TUBA8 and KCNF1 were significantly up-regulated in the model group (P < 0.001), whereas OR10H1 (P < 0.001) and SOAT2 (P < 0.01) was significantly down-regulated (**Figure 11B**). Despite multiple primer design attempts, specific amplification of murine FOLR3 mRNA could not be achieved; This result may be attributed to species-specific differences between FOLR3 in humans and mice, as well as the absence of a clearly defined genetic background in the murine model system; consequently, this study failed to obtain valid validation at the mRNA level.

At the protein level, western blot analysis provided a more comprehensive validation, encompassing all five candidate genes (full-length blots are shown in **Supplementary Figure 1**). The results largely corroborated and extended the qPCR findings. Protein expression levels of KCNF1, OR10H1, and TUBA8 were significantly decreased in the model group (**Figures 11C, D**). Notably, TUBA8 exhibited significant downregulation at the protein level (P < 0.001) despite significant upregulation at the mRNA level. May suggest potential hierarchical regulatory differences during osteoclast differentiation. The protein expression of SOAT2 showed no significant change, consistent with its modest downregulation at the mRNA level. For FOLR3, protein expression showed a notable, though not statistically significant, increasing trend in the model group (**Figures 11C, D**).

## 4 Discussion

Osteoporosis is a prevalent metabolic bone disorder characterized by reduced bone mass and disrupted microarchitecture, leading to an increased risk of fragility fractures. Despite the availability of established diagnostic and therapeutic approaches, current clinical tools remain limited in their capacity to detect early molecular alterations or guide individualized treatment strategies (19, 20). Against this background, the present study integrated rigorous bioinformatics screening with experimental validation to characterize the role of disulfidoptosis-related genes (DRGs) in osteoporosis, yielding three principal contributions. First, a diagnostic model based on three hub genes—SOAT2, FOLR3, and TUBA8—demonstrated moderate accuracy in internal validation and high accuracy in external validation. Second, a striking discordance between mRNA upregulation and protein downregulation was identified for TUBA8, suggesting a previously unrecognized post-transcriptional regulatory mechanism in osteoclast biology. Third, consensus clustering revealed two molecularly distinct osteoporosis subtypes with unique immune infiltration profiles, providing a potential framework for patient stratification. Taken together, these findings advance the understanding of disulfidoptosis in bone metabolism and offer a foundation for precision diagnostic strategies in osteoporosis.

The diagnostic utility of the DRG-based model, achieving an AUC of 0.87 in the internal validation cohort and exceeding 0.9 in the external dataset (GSE230665), underscores the potential of disulfidoptosis-related transcriptomic signatures as diagnostic biomarkers for osteoporosis. Previous bioinformatics and machine learning studies have identified differentially expressed gene panels and constructed predictive models for osteoporosis, generally relying on broad gene sets or focusing exclusively on classical bone metabolism–related transcripts (21). Our targeted selection of DRGs from an established disulfidoptosis-related gene pool represents a methodological advance, aligning mechanistic biological insight with diagnostic utility. Conventional biomarkers, such as bone turnover markers and bone mineral density measured by dual-energy X-ray absorptiometry, provide indirect and often late-stage indicators of skeletal health (22), whereas the present approach links a specific cell death mechanism to disease classification, offering a distinct conceptual and practical basis for biomarker development. Notably, the external validation dataset was derived from femoral tissue while the model was trained on monocyte data; this tissue heterogeneity may contribute to the high AUC value, which should therefore be interpreted as cross-tissue consistency of gene signatures rather than direct generalizability of the diagnostic model. Independent validation in larger, tissue-homogeneous cohorts is therefore warranted before clinical translation.

The most mechanistically significant finding of this study is the profound discordance observed for TUBA8, which was significantly upregulated at the mRNA level yet markedly downregulated at the protein level in the RANKL-induced osteoclast differentiation model. Such mRNA–protein discordance has been documented in other biological contexts and may arise through several post-transcriptional mechanisms (23), including microRNA-mediated translational repression, modulation of mRNA translation efficiency, enhanced proteasomal degradation, or alterations in protein stability under conditions of cellular stress and rapid phenotypic transition. In osteoclastogenesis specifically, RANKL signaling orchestrates extensive cytoskeletal remodeling, and tubulin isoforms are subject to dynamic post-translational modifications—including acetylation, detyrosination, and polyglutamylation—that profoundly influence protein abundance and turnover. The discordance observed for TUBA8 may therefore reflect a compensatory regulatory mechanism in which transcriptional upregulation is counterbalanced by accelerated protein degradation during the cytoskeletal reorganization that accompanies osteoclast differentiation. These interpretations remain speculative in the absence of direct mechanistic evidence, such as protein half-life assays, ubiquitination analysis, or miRNA screening; further functional studies are needed to confirm the underlying regulatory events.

For SOAT2, significant downregulation at the mRNA level was observed without a corresponding change at the protein level, a pattern consistent with post-transcriptional compensation that maintains protein abundance despite reduced transcription. This finding aligns with the established role of SOAT2 in cholesterol esterification and highlights the potential importance of lipid metabolic homeostasis in osteoclast biology (24–26). The apparent maintenance of SOAT2 protein levels may reflect the high demand of differentiating osteoclasts for cholesterol esterification capacity, a hypothesis that warrants direct experimental investigation. For FOLR3, human transcriptomic data identified it as a downregulated differentially expressed gene in osteoporosis; in the murine osteoclast model, protein expression showed a non-significant upward trend, while mRNA-level validation could not be achieved due to species-specific differences between human and murine FOLR3. This discrepancy likely reflects differences in folate metabolic regulatory networks between species, as well as the distinct transcriptional landscape of *in vitro* RANKL-induced osteoclastogenesis compared with the complex *in vivo* pathological environment of human osteoporosis. Given the non-significant nature of the protein trend and the absence of murine mRNA-level validation, conclusions regarding the functional role of FOLR3 in osteoclast differentiation remain preliminary, and its potential relevance as a biomarker or mechanistic target requires further investigation using human-derived experimental systems. Furthermore, the lack of human protein-level validation remains a major constraint for translating these findings to clinical settings. The consistent downregulation of KCNF1 and OR10H1 at both mRNA and protein levels provides more straightforward experimental support for the involvement of these genes in osteoclast differentiation, reinforcing the broader relevance of the DRG signature in bone cell biology, although their precise functional roles in osteoclastogenesis remain to be defined.

CIBERSORT deconvolution analysis revealed distinct predicted immune cell compositional differences between osteoporosis and control samples. Correlation analyses indicated associations between key genes and specific immune cell features—notably, SOAT2 correlated positively with regulatory T cells (Tregs), whereas TUBA8 correlated negatively with resting dendritic cells—suggesting potential crosstalk between disulfidoptosis-related molecular signatures and the immune microenvironment of osteoporotic tissue. These observations are consistent with prior transcriptomic studies implicating immune-inflammatory pathways in osteoporosis progression, including evidence that T cell–derived cytokines promote osteoclastogenesis and that macrophage polarization states influence bone remodeling (27, 28). Our findings extend this literature by linking specific DRGs to predicted immune cell populations, raising the possibility that dysregulation of disulfidoptosis-related pathways contributes to immune perturbation in osteoporosis. However, CIBERSORT applied to monocyte-derived bulk transcriptomes cannot reliably quantify tissue-resident immune cells such as Tregs and dendritic cells; these results are computational estimates rather than direct experimental measurements. Building upon this immunological context, consensus clustering based on the expression profiles of the three hub genes identified two molecularly distinct osteoporosis subtypes—Cluster 1 and Cluster 2—each exhibiting unique transcriptional programs, pathway activity profiles, and predicted immune infiltration characteristics. These subtype-specific immune features suggest a degree of heterogeneity within the osteoporotic immune landscape that may have implications for patient stratification and the development of immunomodulatory therapeutic strategies. It must be emphasized that the subtypes identified here are derived from a relatively small discovery cohort and should be interpreted as hypothesis-generating, requiring prospective validation by flow cytometry, immunohistochemistry, or single-cell transcriptomic approaches in independent and adequately powered clinical cohorts.

Several limitations should be acknowledged. First, the initial DRG list was compiled using a broad keyword search, which may introduce non-specific genes, although multi-step downstream filtering helped reduce false positives. Second, differential expression analysis used a nominal P < 0.05 without FDR correction to retain subtle signals; final conclusions were based on integrated screening rather than raw DEGs. Third, WGCNA relied on a modest correlation threshold (|r| > 0.3) and yielded only five overlapping genes with DRDEGs, indicating limited statistical power that requires validation in larger cohorts. Fourth, bioinformatic analyses were based on human data while experimental validation used murine RAW264.7 cells, with FOLR3 mRNA undetectable in mice due to species differences; human sample validation at the protein level is lacking. Fifth, the mechanism underlying TUBA8 mRNA–protein discordance remains uncharacterized. Sixth, no reproducible R scripts were provided, which may limit full replication of analyses.

Collectively, these constraints indicate that the present study is exploratory in nature and provides a foundational framework for future functional and translational studies.

## 5 Conclusion

This study has successfully developed and experimentally validated a novel diagnostic model for osteoporosis centered on three disulfidoptosis-related hub genes (SOAT2, FOLR3, and TUBA8). Key findings include the identification of complex post-transcriptional regulatory dynamics—most notably for TUBA8, which exhibited significant mRNA upregulation coupled with marked protein downregulation—suggesting a previously unrecognized layer of regulatory control in osteoclast biology. Furthermore, two distinct molecular subtypes of osteoporosis were delineated, each associated with unique immune infiltration profiles, thereby providing a foundation for patient stratification. Significant correlations between these key genes and specific immune cell populations offer new insights into the crosstalk between disulfidoptosis and the bone immune microenvironment.

These results not only highlight the potential for developing a clinically promising diagnostic strategy but also open new research avenues for investigating post-transcriptional regulation and immunomodulatory mechanisms in osteoporosis, paving the way for future precision medicine strategies.

## Data Availability

The original contributions presented in the study are included in the article/**Supplementary Material**. Further inquiries can be directed to the corresponding author.
