# Supplementary material for: Development and experimental validation of an osteoporosis diagnosis model based on disulfidoptosis-related genes and immune infiltration analysis
Source: Front Immunol. 2026 May 28;17:1834059. doi: 10.3389/fimmu.2026.1834059 (PMC13253969; doi:10.3389/fimmu.2026.1834059)
Supplement: Supplementary file 1 [file DataSheet1.zip › Supplementary Material/Supplementary Material.docx]

Supplementary Material

# Supplementary Data

**1.1 Supplementary_code**

# Supplementary Figures and Tables

## 2.1 Supplementary Tables

**Supplementary Table S1**  DRGs_01

**Supplementary Table S2**  DRGs_02

**Supplementary Table S3** DRDEGs

**Supplementary Table S4** MEbrown_genes

**Supplementary Table S5** GO_KEGG

**Supplementary Table S6** GSEA

**Supplementary Table S7** GSVA

## 2.2 Supplementary Figures

**Supplementary Figure 1:**  Western Blot Data
