# Supplementary figures and images for: Development and experimental validation of an osteoporosis diagnosis model based on disulfidoptosis-related genes and immune infiltration analysis
Source: Front Immunol. 2026 May 28;17:1834059. doi: 10.3389/fimmu.2026.1834059 (PMC13253969; doi:10.3389/fimmu.2026.1834059)

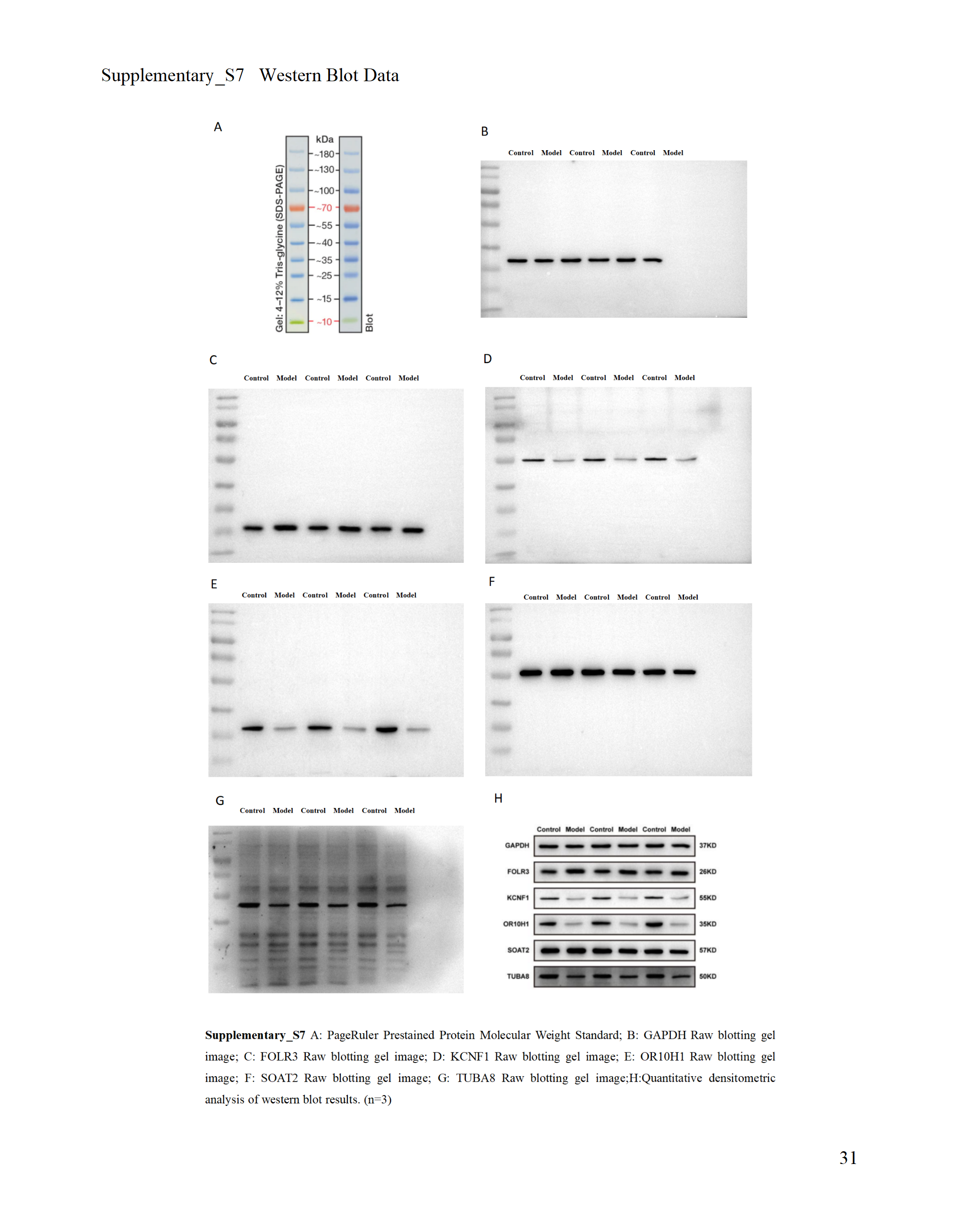

Supplement: Supplementary file 1 [file DataSheet1.zip › Supplementary Material/Supplementary_Figure_1_Western Blot Data.tif]
